# A demographic perspective on longevity and healthspan: Rethinking population ageing through a positive and prospective lens

**DOI:** 10.1111/joim.70151

**Published:** 2026-09-16

**Authors:** Daniele Vignoli, Cosmo Strozza, Agar Brugiavini, Davide Liborio Vetrano, Amaia Calderón‐Larrañaga, Serhiy Dekhtyar, Pietro Grassi, Chiara Seghieri

**Affiliations:** ^1^ Department of Statistics, Computer Sciences, Applications University of Florence Florence Italy; ^2^ Interdisciplinary Centre on Population Dynamics University of Southern Denmark Odense Denmark; ^3^ Department of Economics University of Venice Ca Foscari Venice Italy; ^4^ Aging Research Center Department of Neurobiology, Care Sciences and Society Karolinska Institutet and Stockholm University Stockholm Sweden; ^5^ Stockholm Gerontology Research Center Stockholm Sweden; ^6^ Institute of Management L'EMbeDS, Sant'Anna School of Advanced Studies Pisa Italy; ^7^ Institute of Management L'EMbeDS, Sant'Anna School of Advanced Studies Pisa Italy

**Keywords:** healthspan, longevity, palliative care, population ageing, positive demography, prospective age

## Abstract

Population ageing is portrayed as a threat to the sustainability of health and welfare systems. This narrative—grounded in static chronological definitions of old age—risks overstating the burden of increasing longevity while overlooking changes in survival, health and population composition. In this article, we adopt a demographic perspective to rethink longevity and healthspan through the lens of positive demography, which reframes ageing as a structural transformation that can generate opportunities if institutions, health systems and policies adapt accordingly. Using prospective measures that define old age by remaining life expectancy (LE), we show that population ageing appears less burdensome than conventional indicators suggest (e.g., dynamic dependency ratios fluctuate around 20% compared with 40% under fixed thresholds). Nonetheless, challenges persist, including growing heterogeneity and inequalities in late‐life health and care needs (with healthcare workforce demand by 2071 ranging from a 33% increase to a 16% decrease depending on morbidity trends) and a widening gap between lifespan and healthspan (LE at age 60 has risen at double the rate of healthy LE), resulting in more prolonged dependency and complex end‐of‐life trajectories. Prevention (primordial, primary, secondary and tertiary) and palliative care emerge as crucial but insufficiently integrated components of ageing societies’ health systems. We conclude that a forward‐looking demographic framework—integrating dynamic age thresholds, cohort‐specific health profiles, and equitable access to care across the full continuum—is essential to align societies with realities of longer lives. Population ageing is not a problem to be solved but a context within which health systems and policies must evolve.

## Graphical abstract

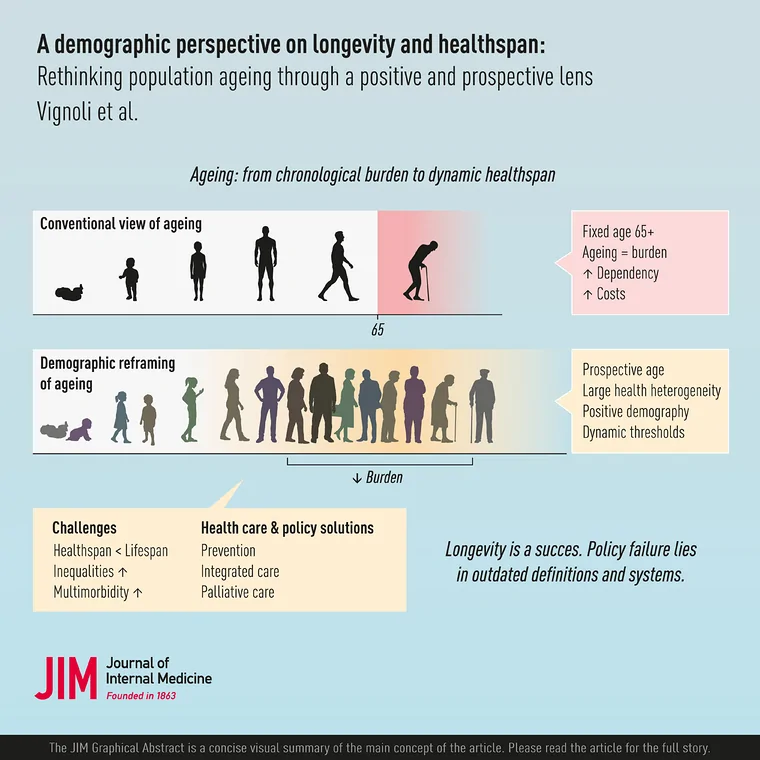

## Introduction

Increases in life expectancy (LE) represent one of the most significant achievements of contemporary societies. Over recent decades, gains in survival at adult and older ages have become the dominant contributor to longevity improvements in high‐income countries. Yet population ageing continues to be framed predominantly as a problem, often associated with fiscal stress, labour shortages and unsustainable health care expenditure. This framing relies heavily on static demographic indicators and fixed chronological definitions of old age, even though established research has shown that such measures do not adequately reflect changes in remaining LE, health and functioning across cohorts [1, 2]. Fixed age thresholds assume equivalence at the same chronological age over time, even as survival improves; they also treat age as a proxy for health and care needs, despite substantial heterogeneity in morbidity and functional capacity in late life [3], thus overstating dependency and the burden associated with population ageing [4]. This growing heterogeneity has also stimulated interest in the concept of biological age, which aims to capture the underlying physiological and functional state of individuals more accurately than chronological age alone. Emerging evidence suggests that individuals of the same chronological age may differ substantially in biological ageing trajectories, vulnerability to disease, and remaining functional reserve, reinforcing the limitations of fixed age‐based definitions of dependency and old age [5, 6].

A demographic perspective suggests that this alarmist framing of ageing is not only incomplete but potentially misleading. Improvements in survival at older ages reflect a long‐term shift in mortality conditions and should not, by definition, be equated with rising dependency [7]. This distinction is often obscured by broader anxieties about demographic change, driven less by ageing itself than by inadequate policy responses to workforce contraction and pressures on welfare systems—especially pensions and health care—as well as by narratives that conflate older age with dependency and decline [8]. Individuals reaching older ages today do so under markedly different conditions than previous generations, both in terms of survival probabilities and functional capacity [9, 10], making chronological age an increasingly unreliable proxy for health status and care needs. These shifts have direct implications for clinical practice and the organization of services for ageing populations.

This article advances a demographic perspective on ageing grounded in the concept of *positive demography* [11], which reframes ageing as a structural transformation that can generate opportunities if institutions, health systems and policies adapt accordingly. In this context, *healthspan*, the portion of life lived in good health and with preserved functional capacity—and not longevity—becomes a central criterion for assessing ageing. This perspective aligns with the emerging field of geroscience, which conceptualizes biological ageing itself as a modifiable driver of multiple chronic diseases and functional decline. By targeting the mechanisms that underlie ageing processes, geroscience shifts attention from the management of individual diseases toward the preservation of function, resilience and healthspan across the life course [12, 13]. This calls for greater attention to how health, functioning and demands for care are distributed across later life, rather than relying on age alone. By scrutinizing the evidence on whether the gains in LE are being accompanied by postponed morbidity and compressed disability, this perspective suggests a rethinking of how ageing should be measured and how the boundaries of old age should be defined. It concludes with a reflection on the implications of a healthspan‐centred approach, from a positive demography perspective, for health systems and policy across the continuum—from prevention to personalized social, medical and end‐of‐life care.

## Positive demography and the reinterpretation of ageing

Positive demography proposes a shift away from deficit‐oriented interpretations of demographic change [11]. It recognizes that contemporary high‐income populations are entering a qualitatively new demographic phase characterized by low fertility, long LE and extended post‐reproductive life [14]. This phase is neither inherently better nor worse than previous ones; its consequences depend on how societies respond [15].

A fundamental distinction in demography is that between ageing ‘from the bottom’ and ageing ‘from the top’, referring to opposite ends of the classical population ‘pyramid’, which over time increasingly resembles a ‘boat’ (stylized in Fig. 1). The former captures the contraction of the base due to sustained low fertility, whereas the latter denotes the expansion of older age groups driven by rising longevity. Although both processes produce older age structures, they are analytically distinct and carry markedly different implications for policy. Ageing from the top, by definition, reflects improvements in survival and living conditions and should therefore be understood as an outcome of population‐level progress rather than an inherent source of risk. Treating population ageing as a homogeneous process obscures these distinctions, downplays the gains associated with increased longevity, and risks overstating the challenges involved. A demographic perspective that fails to differentiate between ageing from the bottom and ageing from the top may inadvertently reinforce alarmist narratives and misdirect policy attention away from the specific mechanisms shaping population age structures and their distinct consequences.

**Fig. 1:**
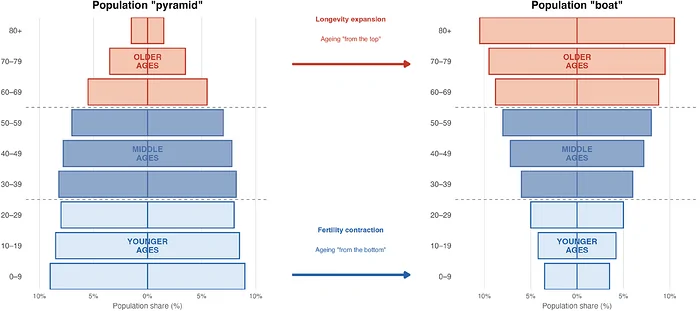
From ‘Pyramid’ to ‘Boat’: Ageing from the bottom and the top.

Along these lines, building on more than a decade of research, Lutz [16] proposes an optimistic reading on human capital and demographic change. He argues that global population decline is not only likely over the long run but may also enhance human wellbeing if driven by universal education [17, 18]. From this perspective, population decline can be reframed as a positive outcome of social development rather than a demographic threat. Central to this perspective is a multidimensional demographic approach that emphasizes population composition by age, sex and education rather than population size alone. Education—especially women's education—simultaneously improves health and economic outcomes and strengthens societies’ adaptive capacity, with demographic differences mattering more for adaptation than mitigation [16, 19, 20]. This approach overcomes the ‘degrowth’ paradigm, emphasizing the role of human capital and sustainable technological improvements to complete a new demographic landscape, especially in the transitional phase [21, 22]. The role of capital—both human and physical—is being reconsidered in the socio‐economic arena. The traditional ‘production function’ is increasingly challenged as the digital (AI) and green transitions demand a profound transformation in skills, knowledge and competencies. New jobs are expected to emerge, replacing those that become obsolete [23]. To fully harness the potential of a workforce that is older, more educated and no longer male‐dominated, both the welfare state and markets must adapt to a changing socio‐economic environment—one in which key life events are no longer confined to narrow, age‐specific windows of opportunity [14].

Several empirical examples illustrate these dynamics. Evidence from the Survey of Health, Ageing and Retirement in Europe data shows that women who were protected during maternity through effective leave provisions display stronger attachment to the labour force and better health at older ages compared to mothers who did not benefit from such policies [24]. As fertile ages extend and increasingly overlap with periods of education and employment, such provisions become even more crucial. At the same time, longer and healthier lifespans require firms to adapt to an older (and more experienced) workforce, redesigning work environments and expanding flexible arrangements such as remote work as a gradual transition between full employment and retirement [25]. Moreover, the traditional dichotomy between ‘leisure time’ and paid market activities is becoming less meaningful [14]. A substantial share of the population is engaged in socially essential but unpaid activities, such as caring for older relatives—effectively providing unpaid welfare services (Grassi et al., 2026, unpublished data). A large proportion of these caregivers are women aged 55–65 and older. Bringing these contributions into view is essential for developing a sustainable reorganization of both society and the economy [26, 27].

## Rethinking old age: From chronological to prospective thresholds

Most demographic and policy‐relevant indicators of ageing rely on fixed age thresholds, typically age 65. This convention assumes stability in health and survival over time and across cohorts, an assumption that is no longer empirically defensible [28]. Fixed thresholds treat improvements in longevity as irrelevant to the definition of old age, thereby inflating measures such as the old‐age dependency ratio and implicitly equating age with need. From a clinical, public health and social perspective this approach is problematic. It risks misaligning health systems and welfare state planning with actual patterns of morbidity and functional limitation.

Recent research challenges the conventional assumption that old age begins at the fixed chronological threshold of 65, arguing instead that ageing should be understood as a relative and multidimensional process shaped by health, functional capacity, socio‐economic context and by inter‐individual differences in biological age, which may diverge substantially from chronological age. Prospective approaches redefine old age based on remaining LE rather than years already lived [29]. Building on early contributions [30, 31, 32, 33], these approaches identify the threshold of old age as the age at which remaining LE falls below a specified number of years, adjusting dynamically to improvements in survival. Empirical applications show that adopting such thresholds substantially attenuates apparent population ageing driven by longevity gains, even though it cannot offset ageing driven by low fertility. Fig. 2 displays a classic demographic indicator—the old‐age dependency ratio—computed as the ratio between the population above a given old‐age threshold and the working‐age population, defined from age 20 up to that threshold. Two thresholds are compared: A fixed chronological threshold at age 65 and a dynamic threshold defined as the age at which remaining LE equals 15 years. Focusing on the thicker lines representing the EU27 averages, the two approaches tell markedly different stories. The fixed threshold portrays a consistently worsening picture, with dependency ratios rising over time to around 40% for women. The dynamic threshold, by contrast, presents a considerably more moderate picture, with ratios fluctuating around 20% over the same period. This divergence illustrates how the choice of age threshold substantially shapes the interpretation of population ageing trends and underscores the analytical value of prospective measures in providing a less alarmist and more demographically grounded assessment.

**Fig. 2:**
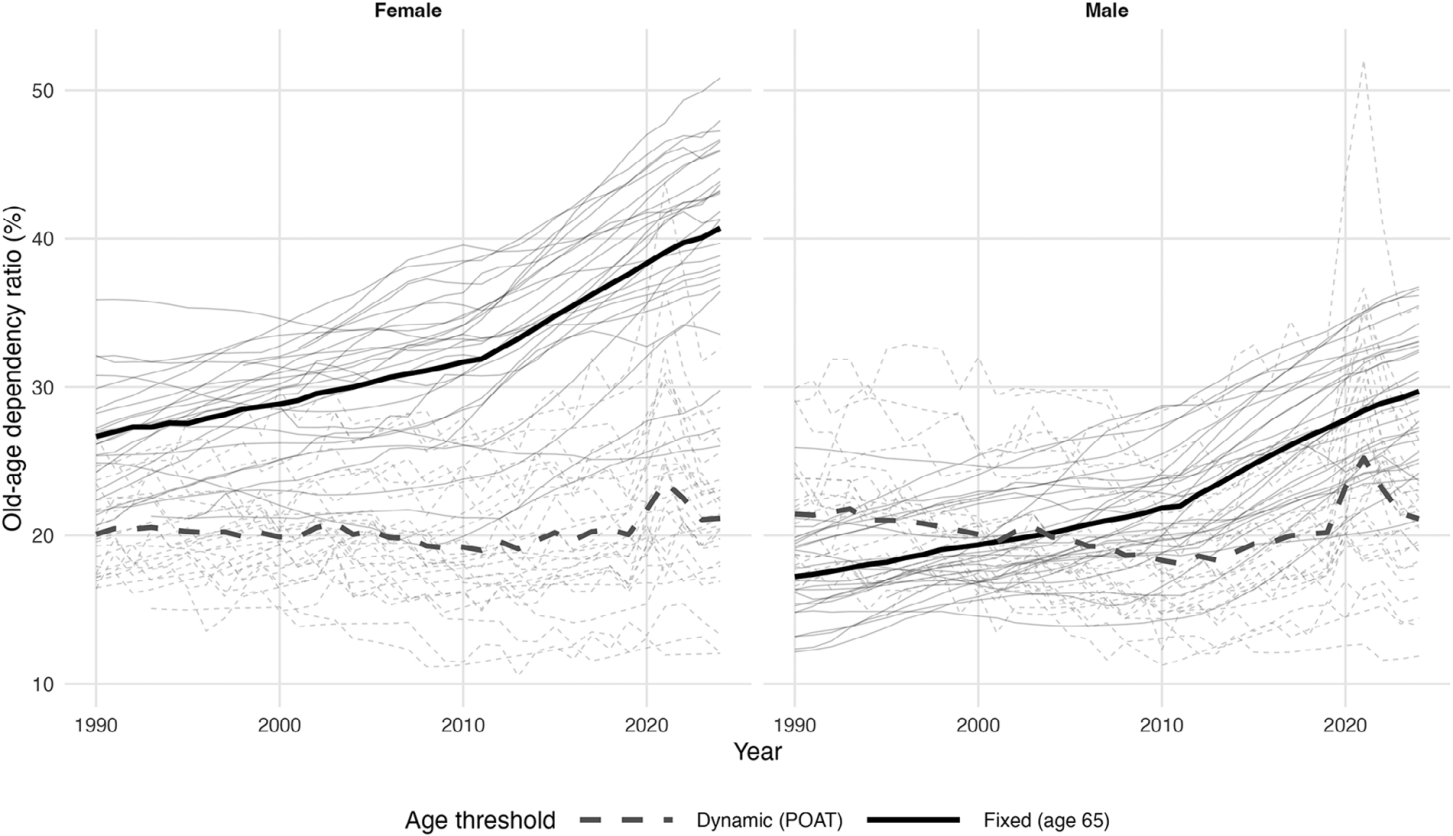
Old‐age dependency ratios under fixed and prospective age thresholds (POAT), EU27 individual countries and averages, 1990–2024. POAT computed using remaining life expectancy of 15 years.

Beyond their demographic implications, fixed age thresholds also have important economic consequences. Pension systems, labour market institutions and fiscal projections often rely on chronological age cut‐offs to define retirement eligibility and dependency, although a number of countries have begun linking statutory retirement age to LE. When longevity increases while retirement ages remain largely unchanged, a growing proportion of life is spent in retirement, placing pressure on pension expenditures and public finances, at least where neither benefit levels nor statutory retirement ages are automatically adjusted to survival gains. However, interpreting these trends solely through the lens of chronological age may overstate the economic burden associated with population ageing. If individuals reach older ages in better health and with greater functional capacity than previous cohorts, many remain capable of contributing economically through paid work, caregiving, volunteering, or other forms of social participation. From this perspective, prospective measures offer a more realistic assessment of the balance between dependency and productive capacity in ageing societies. At the same time, any adjustment of retirement ages must account for substantial socio‐economic inequalities in health and LE, which means that the capacity to extend working lives is unevenly distributed across population groups.

More refined approaches go further by adjusting both the lower and upper age boundaries so as to maintain constant shares of life spent in youth, adulthood and old age. These adjustments typically require only modest shifts in age thresholds compared to the substantial gains in LE, suggesting that a significant portion of the observed ‘ageing from the top’ is driven by outdated age definitions. It should be noted, however, that prospective age does not replace chronological age as a universal metric. Rather, it complements it. Alongside biological age—which captures individual‐level physiological ageing through molecular and clinical markers—prospective age provides a context‐sensitive perspective. These approaches are increasingly relevant within geroscience, which conceptualizes biological ageing as a modifiable determinant of multimorbidity, functional decline, and resilience in later life [34]. Importantly, results based on prospective age depend on the chosen threshold of remaining LE—15 years in this case—and are most informative when interpreted relative to this benchmark, rather than as an absolute definition of old age.

In addition, these adjustments should be accompanied by a coherent revision of the entire life‐course age structure. The potential starting age for employment—here set at 20—may also need to be reconsidered, given the increasing duration of full‐time education. By the same logic, workforce exit can no longer be tied to static retirement ages. Prospective and health‐adjusted measures, increasingly complemented by biomarkers of biological ageing, point to the need to recalibrate thresholds, but simple links to LE risk widening inequalities if health disparities are ignored. To remain both fair and sustainable, welfare states should replace rigid retirement ages with flexible, phased pathways—enabling longer careers for those with high capacity while protecting earlier exits for individuals in poorer health or demanding occupations.

## Postponed mortality, delayed disability and growing heterogeneity in healthspan

Contemporary mortality trends show that survival improvements have increasingly concentrated at older ages. In 27 OECD countries LE at birth exceeded 80 years in 2023, and survival probabilities are increasing [35]. The rapid growth in the number of nonagenarians and centenarians accompanies these shifts. It is worth noting that, despite a deceleration in improvements across several regions of Western Europe since the mid‐2000s, gains in longevity continue, with leading regions persistently pushing the boundaries of human longevity [36].

Mortality risks increase approximately exponentially across most of older adulthood before decelerating at advanced ages [37, 38]. Hence, over time, the mortality schedule has shifted toward later ages. Delayed disability may be one mechanism through which mortality is postponed over time: Individuals reaching extreme old age do so because they benefit from improved health conditions across the life course. As summarized in Table 1, this pattern is observed in several cohort and trend studies, although improvements are more pronounced for some domains of functioning than for others, and trends vary substantially depending on the measure and country considered [39, 40].

**Table 1:** Evidence on cohort and period trends in late‐life functional health and disability.

Study	Population and age‐group	Functional measure	Direction of change and time horizon	Key finding
Beard et al. (2025) [10]	England and China, 60+ years	Intrinsic capacity (index of physical, cognitive, sensory, psychological functions and biological vitality)	Improvement across 1920 and 1950 birth cohorts	Later‐born cohorts entered older age with higher intrinsic capacity. A 68‐year‐old born in 1950 had higher capacity than a 62‐year‐old born 10 years earlier
Falk et al. [9]	Sweden; 75‐year‐olds	ADL^a^ and IADL^b^	Improvement between 1976–77 and 2005–06	ADL disability fell from 13.9% to 5.6% and IADL disability from 33.4% to 13.0%
Crimmins et al. (2009) [41]	USA; Age 70 years	Disability‐free life expectancy	Improvement between 1987 and 1997	Disability‐free life expectancy increased from 10.92 to 11.55 years, whereas disabled life expectancy was unchanged (2.77–2.76 years)
Sundberg et al. (2016) [42]	Sweden; Age 77	Health expectancy (severe disability, mild disability mobility issues)	Somewhat improved between 1992 and 2011	Health expectancy increased (+1.7 years men; +1.1 years women); some indication of expansion of health problems in men
Chatterji et al. (2014) [43]	Review Europe (SHARE^c^), USA (HRS^d^), England (ELSA^e^), others; 60+ years	Chronic morbidity, functioning, disability	Somewhat improved	Evidence for compression especially for disability, longitudinal and high‐income‐country studies. Some evidence for expansion, especially for chronic morbidity
Spiers et al. (2021) [44]	Review of OECD^f^ high‐income countries	Health expectancy trends	Mixed overall pattern; improvement in Sweden	No clear evidence that gains in healthy or disability‐free life expectancy kept pace with gains in total life expectancy; Exception: Sweden, clear improvement for women

^a^ Activities of daily living.

^b^ Instrumental activities of daily living.

^c^ Survey of health, ageing and retirement in Europe.

^d^ Health and retirement study.

^e^ English longitudinal study of ageing.

^f^ Organization for economic co‐operation and development.

Taken together, these findings have major implications for how ageing should be conceptualized. If mortality is postponed and disability delayed, then individuals classified as ‘old’ according to fixed chronological thresholds are not equivalent across cohorts. A 70‐year‐old today faces mortality risks and functional prospects closer to those of a 60‐year‐old several decades ago. Ignoring this fact leads to systematic overestimation of dependency and healthcare needs associated with ageing. Accounting for health and functional capacity in the definition of old age provides a more nuanced picture of population ageing. Traditional indicators, such as the old‐age dependency ratio, tend to overstate the burden of ageing, whereas alternative measures that account for health status—such as health‐adjusted dependency ratios—reveal a substantially lower burden, particularly in countries with better ageing‐related health [45].

Consistent with this perspective, projections of healthcare workforce demand in the EU27 by the European Commission's Joint Research Centre suggest that, under a healthy ageing scenario—where the onset of morbidity is delayed and the burden of age‐related diseases continues to decline—the demographic impact of population ageing can be substantially mitigated [46]. More specifically, whereas demographic ageing alone would require a sizeable expansion of the healthcare workforce—up to +30% for doctors and +33% for nurses by 2071 under a constant disease burden scenario—the continuation of past improvements in population health would reverse this trend. Under a healthy ageing scenario, the projected need for healthcare professionals would instead decline by around 18% for doctors and 16% for nurses relative to 2021 levels. This reflects the fact that, historically, the burden of ageing‐related diseases has been decreasing across all age groups—by approximately 0.9% per year on average since the 1990s—particularly at older ages, thereby compressing morbidity and reducing care needs. In such scenarios, improvements in health at older ages may not only offset but potentially outweigh the effects of population ageing, leading to a more optimistic outlook for the sustainability of healthcare systems and welfare states than suggested by age‐based demographic projections alone. Furthermore, recent analyses highlight that the dividends of this compression of morbidity are not merely organizational but profoundly economic: Promoting healthy ageing and community‐based models has the potential to generate substantial fiscal savings by reducing the intensity of long‐term care needs [47].

Beyond the average improvements in survival and health, however, recent evidence points to growing heterogeneity in later‐life health and a growing polarization in individual frailty and multimorbidity. While overall age‐at‐death variability has declined due to reductions in early‐life mortality, lifespan inequality among those aged 65 and above is increasing globally, suggesting that longevity gains are widening rather than compressing late‐life survival heterogeneity [48, 49]. Research from the United States, the United Kingdom [50] and Italy [51] indicates a stagnation or slight contraction in healthy LE among younger cohorts. Socioeconomic inequalities remain a primary driver of these disparities [52, 53, 54], compounded by the unique epidemiological landscapes that successive birth cohorts have navigated. These patterns reinforce the growing recognition that chronological age alone inadequately captures ageing‐related vulnerability. Individuals and cohorts of the same chronological age may differ substantially in biological age, reflecting cumulative exposures, socioeconomic conditions, lifestyle factors, and disease burden across the life course. Such divergence in biological ageing trajectories may partly explain the widening heterogeneity observed in frailty, multimorbidity, disability and survival at older ages [3, 55]. Most notably, recent cohorts are heavily impacted by the surge in ‘affluent diseases’, such as obesity and metabolic dysfunction [56, 57]. A longitudinal analysis of World Health Organization data (2000–2021) for high‐income countries confirms this divergence. Although both LE and healthy life expectancy (HALE) trended upward until the disruption of the COVID‐19 pandemic, gains in survival have consistently outpaced improvements in functional health [58]. This decoupling is most pronounced in the 60+ cohort, where LE has risen at double the rate of HALE (Fig. 3). Consequently, the bonus years gained by recent cohorts increasingly consist of time spent in moderate‐to‐severe disability. This burden displays a structural gender paradox: Although women maintain a clear survival advantage, they endure a significantly longer absolute period of life with limitations compared to men.

**Fig. 3:**
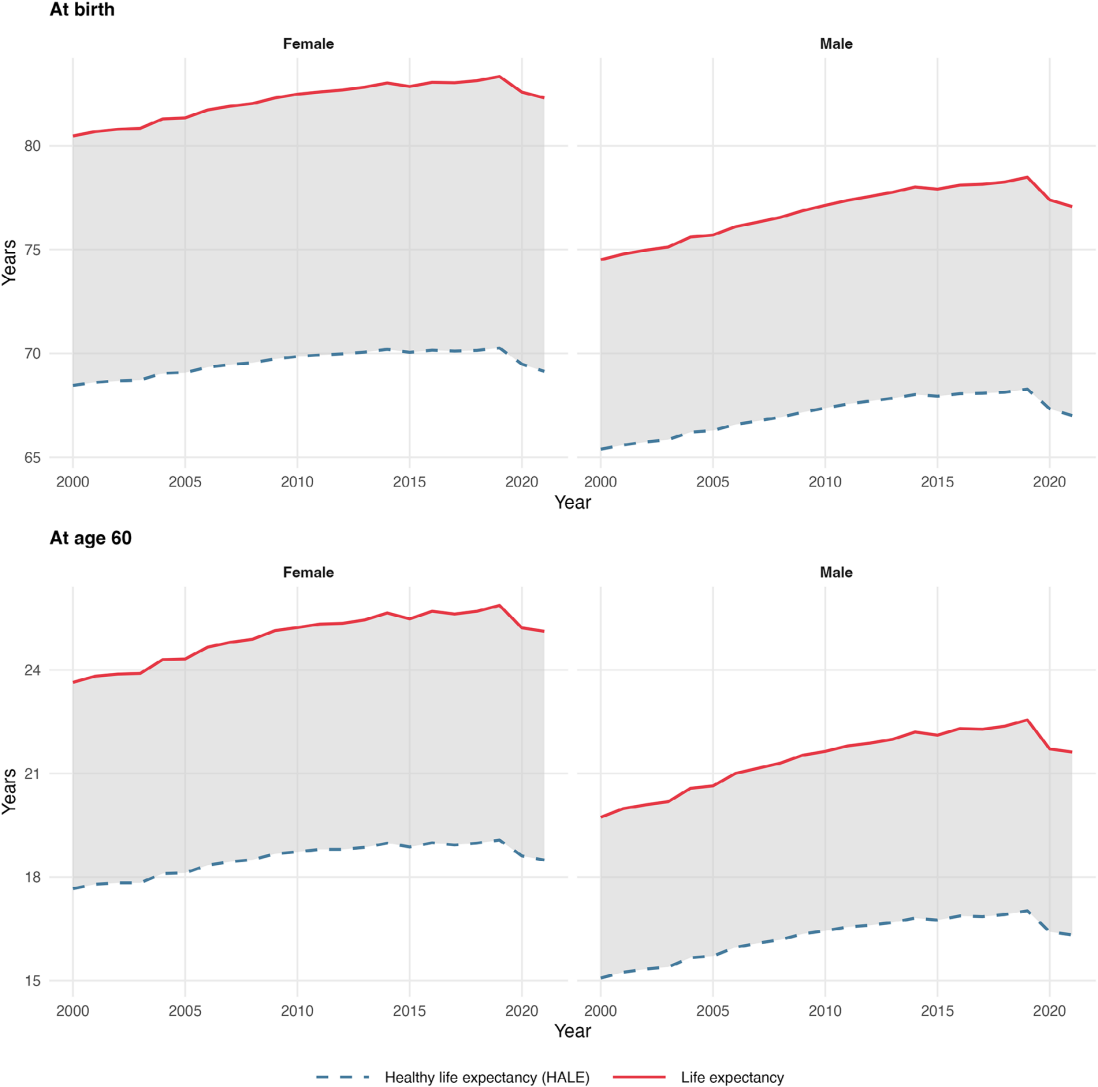
Trends in life expectancy and healthy life expectancy at birth and at age 60 by gender in high income countries (2000–2021). The *y*‐axis shows the expected remaining years of both life and healthy life, and therefore its scale is 65–85 for the former and 15–25 for the latter. WHO Global Health Observatory (2025). High‐income countries (World Bank classification).

Ultimately, this persistent disability gap underscores a central challenge for ageing societies: Longevity gains do not automatically translate into proportional extensions of healthspan. This means that health systems must contend with intensifying demands originating from the complex, accumulated health needs of today's older populations [59]. In this context, biomarker development may offer promise by better estimating one's biological age and enabling earlier identification and risk stratification of major ageing‐related disorders. Recent work suggests that blood‐based biomarkers can help flag older adults with faster accumulation of chronic diseases [55], muscle strength decline [60], cognitive impairment and dementia [61], thereby strengthening early detection and more targeted prevention of several ageing health and functional endpoints. Work in the context of blood‐based biomarkers of Alzheimer's disease pathology is particularly promising, suggesting that such measures may support risk stratification even in general community‐based older populations [62]. At the same time, senolytic treatments that target senescent cells [63] illustrate the promise but also the current limits of a mechanism‐based approach to extending the healthspan, as the evidence in humans remains preliminary, whereas key questions of safety, selectivity and long‐term benefit are unresolved [64]. Taken together, these developments reinforce a healthspan‐centred perspective in which prevention, early detection, and personalized social and medical care are used to delay disease accumulation and functional decline.

## The need for person‐centred integrated care across the full continuum

Increasing longevity has fundamentally altered trajectories of dying. Death is now less often sudden and more often preceded by prolonged periods of decline characterized by multimorbidity, functional dependency and complex symptom burden. This ‘slow dying’ has profound implications for health system organization.

Traditional health care models, largely oriented toward acute and single‐disease interventions, are poorly suited to these extended trajectories. Structured around fragmented and specialized silos, these systems tend to generate discontinuities in care, duplication of interventions, and navigational burden on patients and caregivers [65]. Such fragmentation leads to suboptimal clinical outcomes, reduced treatment adherence and pervasive resource inefficiencies. Moreover, the institutional separation between primary care, specialist services, hospital wards and social care limits the system's capacity to track and manage the evolving complexity of multimorbidity over time [66, 67].

This calls for a shift from disease‐centred and episodic models toward person‐centred integrated care, organized around older individuals’ evolving needs, goals, preferences and life context rather than around single diagnoses or institutional boundaries. However, Swedish evidence suggests that such approaches remain highly heterogeneous and are largely implemented at the micro‐ and meso‐levels, with limited transformation at the system level [68]. Importantly, such models must extend beyond the individual to incorporate family caregivers, who provide a substantial share of care and are themselves increasingly older, creating interdependent care needs that current systems are poorly equipped to address [69].

This systemic inefficiency in managing chronic multimorbidity cascades into the terminal phases of life, creating a growing mismatch between population health profiles and the structure of end‐of‐life care provision. In this context, palliative care has emerged as a central response to the prolonged dying trajectories associated with longevity. Its focus on symptom control, quality of life, and holistic care aligns closely with the needs of ageing populations. However, evidence shows that access to palliative care remains uneven and insufficiently aligned with need [70].

Current literature underscores that access to palliative care is heavily stratified by institutional configurations and diagnostic hierarchies rather than being driven solely by symptom burden. A persistent oncological bias remains evident in healthcare systems, granting cancer patients significantly higher access rates compared to individuals dying from chronic organ failure or neurodegenerative conditions, despite comparable levels of distress [71, 72]. Furthermore, macro‐level analyses reveal profound cross‐national inequalities across Europe, delineating a sharp East–West gradient where service availability and integration are markedly lower in post‐socialist welfare regimes compared to Western counterparts [73, 74].

These patterns indicate that palliative care has not yet been fully integrated into health systems as a standard component of ageing‐related care, contrary to what the WHO claims (World Health Organization. Palliative Care, 2020. Available at: https://www.who.int/news‐room/fact‐sheets/detail/palliative‐care). Instead, it remains attached to specific diagnoses and institutional pathways, limiting its capacity to respond to the realities of prolonged, heterogeneous dying trajectories [75]. From a demographic perspective, this represents a critical failure of adaptation. Longevity gains achieved through decades of improvements in survival risk being undermined if end‐of‐life care remains fragmented and inequitable.

## Conclusion

Population ageing driven by postponed mortality reflects demographic success, yet its benefits can only be fully realized if institutions adapt to the changing relationship between age, health and care needs. The key limitation in much of the debate on population ageing is treating it as a static condition rather than a dynamic process operating at multiple levels. At the individual level, ageing unfolds continuously across the life course, through gradual changes in health and functional capacity. At the population level, however, these trajectories are constantly reshaped by cohort replacement and by broader forces—medical innovations, pandemics, wars and climate change—that alter how, when, and where people age. Ageing is, therefore, not a fixed state but a lifelong, dynamic and heterogeneous process, in which both individual variability and shifting historical contexts play a central role. In this context, chronological age alone becomes an increasingly limited descriptor of ageing‐related vulnerability, whereas concepts, such as biological age, may better capture differences in physiological reserve, resilience and functional trajectories across individuals and cohorts.

A demographic perspective on longevity and healthspan points to the need for structural rather than incremental adjustments in health policy. First, ageing metrics used for planning and evaluation should move beyond static chronological thresholds and incorporate prospective, health‐adjusted measures. Dynamic measures of ageing may also contribute to aligning social policies with contemporary populations. This shift would provide a more accurate basis for anticipating care needs and allocating resources. Second, health policy must adopt a life‐course perspective. Emerging insights from geroscience further support this perspective by suggesting that the biological processes underlying ageing may be modifiable and shared across multiple chronic conditions. This shifts attention from the isolated treatment of individual diseases toward preserving function, resilience, and healthspan throughout the life course. Longevity and healthspan reflect cumulative exposures and investments across decades. Prevention, lifestyle interventions and reduction of social inequalities are essential to sustain health gains into old age. Third, person‐centred integrated care across the full continuum—from prevention to end‐of‐life care—should be mainstreamed as an integral component of health systems in ageing societies. This requires moving away from diagnosis‐based referral models toward needs‐based approaches that recognize multimorbidity and frailty as central features of late‐life health.

A positive and prospective demographic framework reveals that much of the perceived burden of ageing stems from outdated definitions and misaligned systems rather than from longevity itself. Redefining old age, acknowledging heterogeneity in biological and functional ageing trajectories, and ensuring equitable access to coordinated social, medical and end‐of‐life care are essential steps toward aligning health systems with the realities—and the desirability—of longer lives. Population ageing is not a problem to be solved but a context within which health systems and policies must evolve.

## Author contributions

**Daniele Vignoli**: Conceptualization; writing—original draft; writing—review and editing; supervision. **Davide Liborio Vetrano**: Conceptualization; writing—review and editing. **Agar Brugiavini**: Conceptualization; writing—review and editing. **Amaia Calderón‐Larrañaga**: Conceptualization; writing—review and editing. **Chiara Seghieri**: Conceptualization; writing—review and editing. **Cosmo Strozza**: Conceptualization; methodology; data curation; writing—review and editing; formal analysis. **Serhiy Dekhtyar**: Conceptualization; writing—review and editing; methodology. **Pietro Grassi**: Conceptualization; writing—review and editing; data curation.

## Conflict of interest statement

The authors declare no conflicts of interest.

## Funding information

**Daniele Vignoli** and **Agar Brugiavini** acknowledge the funding from the European Union–Next Generation EU, in the context of The National Recovery and Resilience Plan, Investment Partenariato Esteso PE8 ‘Conseguenze e sfide dell'invecchiamento’, Project Age‐It (Ageing Well in an Ageing Society, PE8‐B83C22004800006).

## AI disclosure

We acknowledge the use of Chat‐GPT (version 4.0, June 2026) as well as Gemini (version 3.1, June 2026) to assist us with general text editing, while the scientific content is entirely those of the authors. All AI‐assisted text was reviewed and revised by the authors to ensure accuracy and clarity of meaning.
